# Nut intake and stroke risk: A dose-response meta-analysis of prospective cohort studies

**DOI:** 10.1038/srep30394

**Published:** 2016-07-29

**Authors:** Chuan Shao, Hui Tang, Wei Zhao, Jianquan He

**Affiliations:** 1 Department of Neurosurgery, Nanchong Central Hospital (The Second Clinical College of North Sichuan Medical College), Nanchong, Sichuan, China

## Abstract

We aim to quantify the effects of nut intake on risk of stroke by a dose-response meta-analysis with a random-effects model. Two databases (PubMed and Emabse) were searched for prospective cohort studies regarding nut intake and stroke risk. Studies were included if they fulfilled the predefined criteria. Eleven articles encompassing fourteen cohort studies were included in final analysis. The pooled relative risk (RR) of stroke for the highest versus (vs.) lowest category of nut intake was 0.88 (95% confidence interval [CI] 0.80-0.97). The power to detect a RR of 0.88 for the highest versus vs. lowest category of nut intake was 86.2%. In multiple subset analyses by gender, location, and stroke subtype, the inverse association was only found in women (RR = 0.84, 95% CI 0.73–0.96) and Asia (RR = 0.79, 95% CI 0.67–0.93). In the dose-response meta-analysis, evidence for a nonlinear association between nut intake and stroke risk was observed and a RR of 0.86 was conferred for 12 g/day. Based on the Grading of Recommendations Assessment, Development, and Evaluation (GRADE) system, the quality of evidence was moderate. In conclusions, finding from current meta-analysis of fourteen cohort studies indicates that nut intake may be related to decreased risk of stroke.

Stroke remains the second major cause of death and the third most common cause of disability worldwide^1^. It has been estimated that approximately 12 million people die from stroke and stroke accounts for more than 200 million disability-adjusted life-years lost globally in 2030^1^. Concerning the various burden caused by stroke is dire, effective preventive measures are warrant to be implemented. To date, there are few known preventive strategies. Cigarette smoking, alcohol, hypertension, and diabetes mellitus are the well-known modifiable risk factors for stroke. In recent years, dietary factors have aroused particular attention in epidemiologic studies. For dietary nut intake, its preventative effects on the pathogenesis of cardiovascular diseases, type 2 diabetes, and certain cancers have been shown in observational and biological studies^2^,^3^,^4^,^5^. Therefore, it is possible that nut intake may confer a lower risk of stroke. The relation between nut intake and stroke has been investigated in a number of prospective cohort studies, showing inconsistent results^6^,^7^,^8^,^9^,^10^,^11^,^12^,^13^,^14^,^15^,^16^,^17^,^18^,^19^. In 2014, two meta-analyses of four cohort studies suggested nut consumption was not associated with risk of stroke^2^,^20^. Another meta-analysis of six cohort studies published before June 2014 indicated that nut consumption was inversely associated with risk of stroke^21^. Actually, overlapped data^7^,^9^,^12^ were included in previous meta-analysis by Zhang *et al*.^21^. Furthermore, seven additional cohort studies with large simple sizes have evaluated this association^13^,^14^,^15^,^16^,^17^,^18^,^19^. Thus, no definite conclusion can be drawn from previous meta-analyses. There we performed an updated meta-analysis with a dose-response approach.

## Methods

The present meta-analysis was performed according to the proposal for Preferred Reporting Items for Systematic Reviews and Meta-Analyses (PRISMA) guidelines^22^. Since all included studies were published officially, ethical approval was not necessary. There was no registered protocol.

### Search Strategy

PubMed (http://www.ncbi.nlm.nih.gov/pubmed/) and Embase (http://www.embase.com/) databases were searched in Feb. 14, 2016 with the following medical subject headings or keywords: “stroke”, “cerebrovascular diseases”, “cerebrovascular disorder”, “cerebrovascular accident”, “nut”, “diet”, “lifestyle, “risk”, “risk factors”, and “risk assessment”. The detail of search strategy was shown in Supplementary Table 1. References cited in retrieved articles were also reviewed.

### Selection Criteria

When the initial search was performed, we deleted duplicate records. A second screen of the titles and abstracts was done to assess relevance. Finally, we reviewed full-text articles to determine whether the studies were included in final analysis or not.

Published prospective cohort studies fulfilling the following criteria were identified: (1) assessing the relationship between nut consumption and risk of stroke; (2) providing estimates of the relative risk and corresponding confidence interval, or other information sufficient to calculate them. If more than one report from the same study were published, the one with the longest years of follow-up were included in the present analysis.

### Data Extraction

The data were extracted by one reviewer and examined independently by another two reviewers. Any discrepancies were resolved by discussion. The available data from each included studies included first author, country where the study was performed, cohort name, sex, people’ age at enrollment, years of follow-up, number of cases observed, methods for the measurement of nut intake, number of total participants, methods of stroke diagnosed, most-adjusted multivariable RR with corresponding 95% CI for the different categories of nut intake (when available, the number of cases and non-cases for each exposure level were also collected), and confounders controlled in adjusted models.

### Assessment of Methodological Quality

We used the 9-star Newcastle-Ottawa Scale (Available online: http://www.ohri.ca/programs/clinical_epidemiology/oxford.asp accessed Feb 14, 2016) to assessed methodological quality. The Newcastle-Ottawa Scale judges a study on the basis of selection of study groups, comparability of groups and ascertainment of the exposure or outcome of interest for case-control or cohort studies respectively. Due to no unified standards well established, we defined that studies with ≥6 stars were considered as at low risk of bias in our study.

### Grading Quality of Evidence

The GRADE system (GRADEpro, version 3.6.1) was used to assess the quality of evidence. Evidence from cohort studies begins with a grade of “Low”. The quality of evidence was upgraded for large magnitude of effect, plausible residual confounding that would not reduce the effect size, and a dose response gradient, or downgraded for inconsistency, indirectness, imprecision, and publication bias^23^. Finally, the quality evidence is categorized as high, moderate, low, and very low.

### Statistical Analysis

The RR with corresponding 95% CI was used as the effect size for all studies and the hazard ratios were directly considered as RRs. This method has been widely used in systematic review and/or meta-analysis^2^,^5^,^21^,^24^,^25^,^26^,^27^. Studies only providing results stratified by sex (male vs. female) or stoke subtypes (ischemic stroke vs. hemorrhagic stroke) were treated as two separate reports^25^. We pooled RR with 95% CI for the highest vs. lowest levels to assess the association between nut consumption and risk of stroke with the DerSimonian and Laird random effect model^28^, as this approach considers the variation of within-study and between-study. Subgroup analyses were performed by gender (male vs. female), location where the study was performed (United States vs. Europe vs. Asia), stroke subtypes (ischemic stroke vs. hemorrhagic stroke), and time of follow-up (>10 vs.<10 years). One study was excluded each time from sensitivity analysis to assess the influence of the individual data set on the overall result. Publication bias was evaluated by visually inspecting a funnel plot and Egger test^29^.

We further quantified dose–response relationships of nut consumption and risk of stroke based on the method described by Greenland and Orsini N^30^,^31^. To conduct this analysis, intake in servings per day was converted into grams/day using the standard conversion (1 serving = 28 g)^3^,^19^. This method requires that the number of cases and person-years (non-cases) for at least three level of exposure were reported. Also, the adjusted RR with corresponding 95% CI or its standard error for each median/mean level of exposure should be shown in original studies. When ranges of nut intakes were reported, the midpoint of the range was used. When the highest category was open ended, we assumed the midpoint of the category was set at 1.2 times the lower boundary. When the lowest category was open ended, we set the lower boundary to zero. We used restricted cubic splines with three knots at percentiles 20%, 50%, and 80% of the distribution to evaluate a potential curve linear association between nut exposure and stroke. A P value for curve linearity or non-linearity was calculated by testing the null hypothesis that the coefficient of the second spline is equal to zero.

Homogeneity across studies was tested by Q statistic and quantitatively measured by the I^2^ statistic^32^,^33^. For Q statistic, a significant heterogeneity was defined as a P value < 0.10. The values yielded by I^2^ test range from 0 to 100%. In our study, values of the I^2^ statistic < 25% are representative of low heterogeneity and those > 75% of high heterogeneity. Correspondingly, the value ranging between 25 and 75% represents moderate heterogeneity.

All statistical analyses listed above were done with STATA 12.0 software (StataCorp, College Station, TX). P values less than 0.05 were considered statistically significant, except where otherwise specified.

Power calculation with the method proposed by Cafri *et al*.^34^ was conducted by SAS version 9.3 (SAS Institute Inc, North Carolina, USA).

## Results

### Literature Search

The flow diagram of screened, excluded, and included studies was shown in Supplementary Figure 1 and the reasons for excluded studies were that the studies were published as news, letters, comments, reviews, meta-analyses, or conference abstracts; involved dietary patterns; and had shorter follow-up than others on the same cohort (Supplementary Table 2). Ten articles were identified from database search^6^,^8^,^10^,^12^,^13^,^14^,^15^,^16^,^17^,^19^. One additional study was found in references cited in retrieved articles^18^. Of 11 articles, two shared a small number of subjects^8^,^15^. Therefore, we included both of the studies and excluded one from sensitivity analysis. Finally, eleven articles were included^6^,^8^,^10^,^12^,^13^,^14^,^15^,^16^,^17^,^18^,^19^.

### Study Characteristics

Supplementary Table 3 shows the main characteristics of the included studies. A total of eleven articles were eligible for this study, comprised of fourteen cohort studies. These studies were published between 2000 and 2015. Eight cohort studies were recruited in United States, two in China, one in Australia, one in Italy, one in the Netherland, and one in German. The age of subjects at baseline ranged from 30 to 86.7 years. The follow-up duration ranged from 4.4 to 30 years. Most of studies used death from stroke as the endpoint, except for four studies, in which total cases from both stroke incidence and death were included^8^,^10^,^13^,^19^. Data on nut intake was ascertained by a Food Frequency Questionnaire (FFQ). The study-specific risk estimates for the highest vs. lowest levels of nut intake ranged from 0.47 to 1.37, and none of which reached statistically significant. A great number of potential confounders, such as age, race, body mass index (BMI), gender, waist-hip ratio (WHR), blood pressure (BP), smoking, alcohol intake, education level, physical activity, history of diabetes, myocardial infarction, hypercholesterolemia, and cancer, intakes of cholesterol, saturated fat, meat, multivitamin, carotenoids, dietary fiber, whole grains, and fruit/vegetable, and total energy, and menopausal status and hormone use (in women), were taken into account.

### Study Quality

Supplementary Table 4 shows the results of study quality assessment in detail. All studies were awarded six stars or more, suggesting the included studies were categorized as at low risk of bias.

### Primary result: High vs. Low Intake Analyses

**Figure 1:**
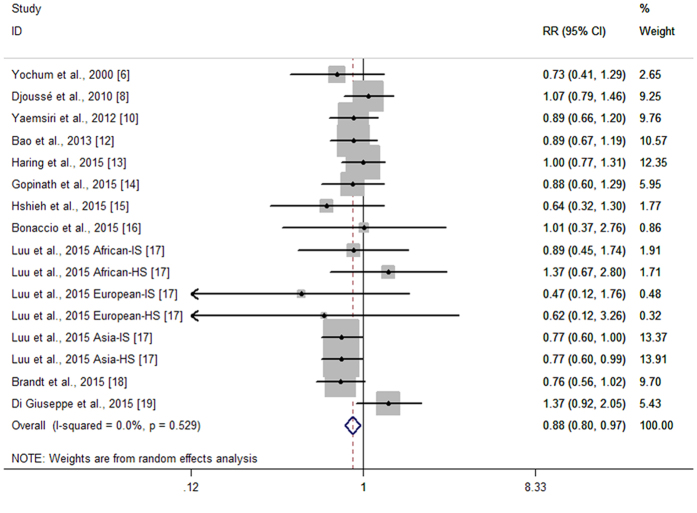
Forest plot of nut intake and stroke risk.

Fourteen cohort studies from eleven articles reported the association between nut intake and stroke risk^6^,^8^,^10^,^12^,^13^,^14^,^15^,^16^,^17^,^18^,^19^. Figure 1 shows the study-specific RRs with 95% CIs and the overall result. The summary RR of stroke risk for the highest versus lowest categories of nut intake was 0.88(95% CI 0.80–0.97). No statistically significant heterogeneity across studies was found (P = 0.529, I^2^ = 0%). Seven of the eleven articles addressed the association between nut intake and stroke mortality^6^,^12^,^14^,^15^,^16^,^17^,^18^,^19^. The summary RR for stroke mortality with nut intake was 0.81(95% CI 0.72–0.91), without statistically heterogeneity across studies (P = 0.966, I^2^ = 0%).

### Subset Analyses

#### Sex

Nine articles provided sufficient data for subgroup analysis^6^,^8^,^10^,^12^,^13^,^14^,^15^,^17^,^19^. The summary RR of stroke in females was 0.84(95% CI 0.73–0.96), without statistically heterogeneity (P = 0.610, I^2^ = 0%). The corresponding estimate for males is 0.97(95% CI 0.81–1.16), with moderate statistically heterogeneity (P = 0.155, I^2^ = 29.6%).

#### Geographic Area

Of the fourteen cohort studies from eleven articles, eight were recruited in United States^6^,^8^,^10^,^12^,^13^,^15^,^17^, three in Asia^14^,^17^, and three in Europe^16^,^18^,^19^. The summary RRs were 0.93(95% CI 0.82–1.06) for studies conducted in United States, 0.79 (95% CI 0.67–0.93) for studies in Asia, and 1.0 (95% CI 0.64–1.57) for studies in Europe. There was no statistically heterogeneity (United States: P = 0.798, I^2^ = 0%; Asia: P = 0.825, I^2^ = 0%), but moderate statistically heterogeneity was observed for Europe (P = 0.069, I^2^ = 62.6%).

#### Stroke Subtype

Five articles provided data on ischemic stroke^8^,^10^,^13^,^17^,^19^. The summary RR of ischemic stroke was 0.95(95% CI 0.79–1.14), with moderate statistically heterogeneity (P = 0.131, I^2^ = 39.2%). Three articles provided data on hemorrhagic stroke^8^,^13^,^17^. A RR of 1.09(95% CI 0.73–1.63) was given in combined analysis of these studies. Also, moderate statistically heterogeneity was shown (P = 0.090, I^2^ = 50.2%).

#### Years of Follow-up

The summary RR was 0.87(95% CI 0.77–0.97) for those with a median follow-up of ≥10 years^6^,^8^,^12^,^13^,^17^,^18^, without statistically heterogeneity (P = 0.587, I^2^ = 0%). Among the cohort studies with less than ten years of follow-up^15^,^16^,^17^,^19^, the pooled RR was 0.92(95% CI 0.75–1.12), with low statistically heterogeneity (P = 0.394, I^2^ = 4.9%).

### Sensitivity Analysis

**Figure 2:**
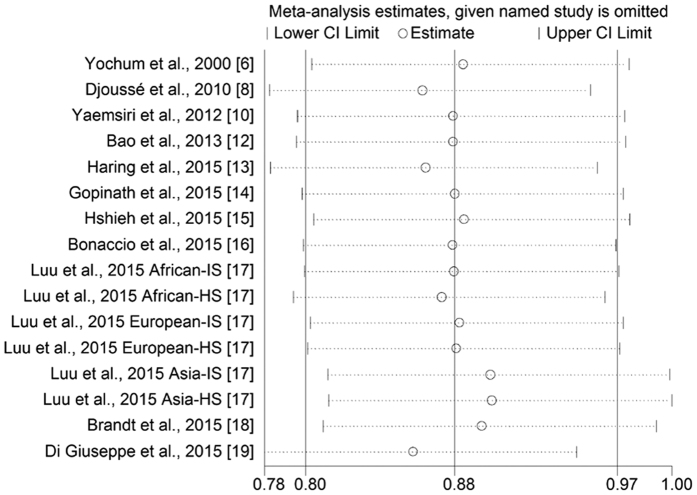
Sensitivity analyses of nut intake and stroke risk.

The influence of single study on overall result was estimated (Fig. 2). A borderline significant association was observed after excluding the Shanghai Women’s Health Study and Shanghai Men’s Health Study (RR = 0.90, 95% CI 0.82–1.00; Supplementary Table 5).

### Publication Bias

**Figure 3:**
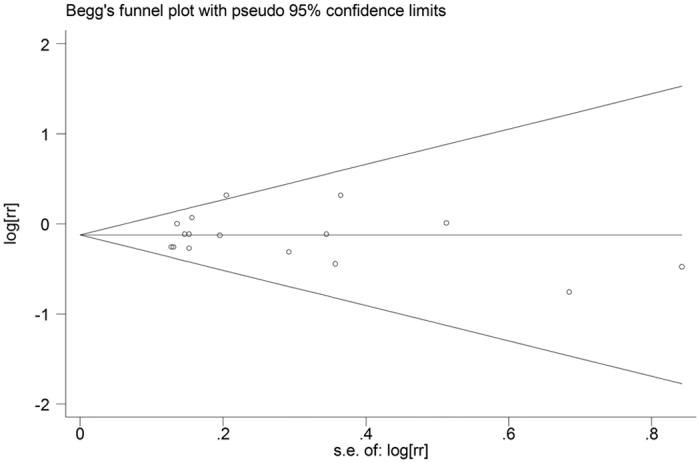
Funnel plot of nut intake and stroke risk.

The funnel plot appears to be symmetrical, indicating that there is no evidence for publication bias (Fig. 3). Also, this evidence was strengthened by formal statistical test (P for Egger’s test = 0.932).

### Dose-Response

**Figure 4:**
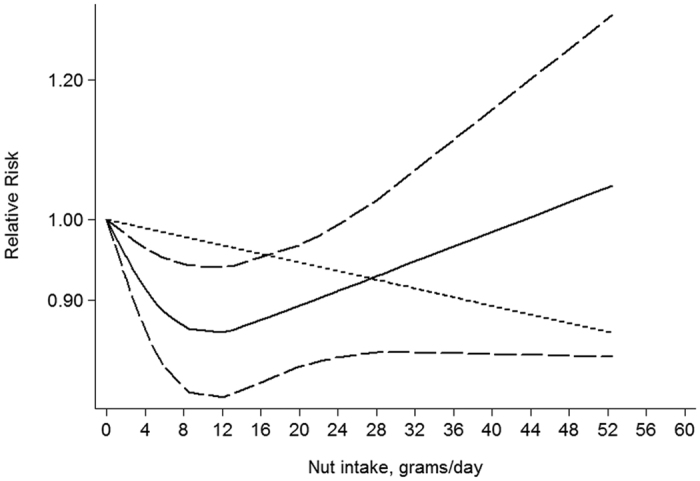
Dose-response analysis of nut intake and stroke risk.

Seven articles were identified in dose-response analysis^12^,^13^,^14^,^15^,^17^,^18^,^19^. A nonlinear association between nut intake and stroke risk was observed (P = 0.026, Fig. 4). The estimated RR was 0.86(0.79–0.94) for 12 grams of nut per day.

### Power Analysis and Quality of Evidence

The power was 86.2% to detect a RR of 0.88 for highest versus lowest category of nut intake (Supplementary Appendix 1). On the basis of the Grade system guideline, the quality of evidence was moderate (Supplementary Table 6).

## Discussion

Our meta-analysis of 14 prospective cohort studies from eleven publications provides evidence that nut intake is inversely associated with risk of stroke and a linear association between nut intake and stroke risk did not exist.

Nut contains a variety of nutrients with anti-inflammatory, antioxidant, and anticarcinogenetic properties, involving the unsaturated fatty acids, folate, niacin, vitamin E, and vitamin B-6, dietary fiber, phytoestrogens, and micronutrients (i.e. copper, magnesium, potassium, calcium, and zinc)^35^,^36^. Furthermore, some evidences for beneficial effects of nut intake on vascular reactivity have been observed^35^. Thus, the reason why nut intake was considered as a protective factor for stroke might be hypothesized.

In subgroup analyses by gender and geographic area, significantly inverse association between nut consumption and stroke risk was only observed for female and Asia subsets. Common explanations for these discrepancies are (1) different dietary habits and genetic backgrounds were shared, and (2) the chance results were identified, as a small number of studies were included in the subgroup analyses.

Hemorrhagic and ischemic strokes vary in pathogenesis and prognosis. With regard to the incidence, ischemic strokes are responsible for about 87% of all cases. However, hemorrhagic strokes account for about 40% of all stroke deaths, although hemorrhagic strokes are less common. Moreover, some studies showed people bear different risk from the same risk factor^37^. These disparities may suggest the benefit effect of nut consumption varies in outcome of stroke and stroke subtypes. In our meta-analysis, an overwhelming majority of cases were the death from stroke and limited evidence for stroke subtypes was eligible. Therefore, our subgroup results, of an increased risk in hemorrhagic strokes but a decreased risk of ischemic strokes with nut consumption, should be interpreted with caution and detailed datasets on stroke subtypes and endpoint are warranted to confirm this finding.

Up to now, it remains unclear that whether different types of nuts have different effects on stroke. To our knowledge, there only two articles performed separate analyses of the consumption of peanuts and tree nuts with stroke risk^12^,^18^. In the Netherlands Cohort Study of 120,852 men and women aged 55–69 years at baseline, inverse association was observed for tree nuts (RR = 0.74) and peanut butter (RR = 0.86), although the results did not reach significance^18^. Another analysis of 76,464 women and 42,498 men with more than 24 years of follow-up shows similar results for the two types of nuts in model of two or more times per week versus never consumption (peanuts: RR = 0.97, 95% CI 0.67–1.40; tree nuts: RR = 0.96 95% CI 0.78–1.19)^12^. For other nuts consumption, such as walnuts, almonds, brazil nuts, cashews, macadamia nuts, and hazelnuts, no data were provided. The nutrient content of nuts varied^35^. For example, walnuts are much richer in alpha-linolenic acid, polyunsaturated fatty acids, and linoleic acid than peanuts^35^. However, peanuts contain more monounsaturated fatty acids, protein, niacin and potassium^38^. In addition, chestnuts contain little fat, but up to 75.8% nutrient is fat in macadamia^35^. Therefore, the anti-inflammatory and antioxidant capacity of each type of nuts may be different and its preventable effect on stroke risk needs to be clarified in future studies.

The major concern about safety of nut consumption is possible weight gain^35^. Nuts are energy-dense foods with high-fat content. In general, frequent nut consumption may promote unwanted weight gain, which, in turn, increases risk of many chronic degenerative diseases. However, there is considerable scientific evidence that frequent ingestion of nuts did not have adverse effects on energy balance or body weight, as reviewed previously^35^,^39^,^40^. The potential mechanistic hypotheses accounting for the null effect on body weight mainly includes (1) nuts were dominated by unsaturated fats; (2) a high supply of fiber and vegetable proteins in nuts increases satiety ratings; (3) some substitutions of nuts could improve insulin sensitivity; and (4) other mechanisms, such as the structure of lipid-storing granules, various fiber components, tocopherols, phytosterols, and phenolic compounds have been discussed in detail in a previous review^39^. Therefore, based on current evidence, nuts are safe to consume.

Several meta-analyses of the relationship between nut intake and stroke risk have been published. The discrepancies between previous meta-analyses and ours should be noted (Supplementary Table 7). A major strength is that we included fourteen cohort studies from 11 publications. And thus, a great number of subjects were included in present meta-analysis than previous meta-analyses. In addition, the power analysis was not performed in previous meta-analyses. Our analysis shows the power was 86.2% to detect a RR of 0.88 for highest versus lowest category of nut intake, indicating the statistical power was enough to detect such a significant finding. Another is that the quality of evidence is assessed according to GRADE methodology.

Like others of similar design, our met-analysis has some limitation. First, the effect of residual confounders on the association cannot be elucidated utterly, although we used the most-adjusted RRs. Second, some included studies reported data about total nuts and peanut butter, and thus an underestimation of the risk with the true amount of nut consumed may be resulted out. Third, the issue whether the relationship between nut intake and stroke risk varies by the prepared method was not addressed, as none of included studies provided information. Fourth, potential publication bias is an intimidation to robustness of our findings, although there was no evidence for publication bias.

Our meta-analysis of published prospective cohort studies supports nut intake is associated with a reduced risk of stroke. The risk of stroke with nut intake seems to be lower in Asian. The reduced risk was pronounced at the level of 12g/gay. The quality of evidence was moderate. This finding supports daily nut intake is an important part of healthy lifestyles and could be recommended in the prevention of stroke.

## Additional Information

**How to cite this article**: Shao, C. *et al*. Nut intake and stroke risk: A dose-response meta-analysis of prospective cohort studies. *Sci. Rep.* **6**, 30394; doi: 10.1038/srep30394 (2016).

## Supplementary Material

Supplementary Information
