# Supplementary material for: Nut intake and stroke risk: A dose-response meta-analysis of prospective cohort studies
Source: Sci Rep. 2016 Jul 29;6:30394. doi: 10.1038/srep30394 (PMC4965766; doi:10.1038/srep30394)
Supplement: Supplementary Information [file srep30394-s1.pdf]

Nut intake and stroke risk: A dose-response meta-analysis of prospective cohort studies

Chuan Shao, Hui Tang, Wei Zhao, Jianquan He

Supplementary Table I Database search strategy and results

Queries in PubMed				Queries in Embase			
Search	Query	Items found	Data	Search	Query	Items found	Date
#14	Search #5 AND #9 AND #13	4396	2016/2/14	#14	Search #5 AND #9 AND #13	9745	2016/2/14
#13	Search #10 #11 OR #12	1866877	2016/2/14	#13	Search #10 #11 OR #12	2606133	2016/2/14
#12	Search risk assessment	336131	2016/2/14	#12	Search 'risk assessment'/de OR 'risk assessment'	397066	2016/2/14
#11	Search risk factors	969445	2016/2/14	#11	Search 'risk factors'/de OR 'risk factors'	865784	2016/2/14
#10	Search risk	1866876	2016/2/14	#10	Search 'risk'/de OR 'risk'	2606133	2016/2/14
#9	Search #6 OR #7 OR #8	519603	2016/2/14	#9	Search #6 OR #7 OR #8	682212	2016/2/14
#8	Search lifestyle	119318	2016/2/14	#8	Search 'lifestyle'/de OR 'lifestyle'	139640	2016/2/14
#7	Search diet	414016	2016/2/14	#7	Search 'diet'/de OR 'diet'	564329	2016/2/14
#6	Search nut	7945	2016/2/14	#6	Search 'nut'/de OR 'nut'	13764	2016/2/14
#5	Search #1 OR #2 OR #3 OR # 4	426474	2016/2/14	#5	Search #1 OR #2 OR #3 OR # 4	403749	2016/2/14
#4	Search cerebrovascular accident	246433	2016/2/14	#4	Search 'cerebrovascular accident'/de OR 'cerebrovascular accident'	236641	2016/2/14
#3	Search cerebrovascular disorder	306112	2016/2/14	#3	Search 'cerebrovascular disorder'/de OR 'cerebrovascular disorder'	51874	2016/2/14
#2	Search cerebrovascular diseases	312454	2016/2/14	#2	Search 'cerebrovascular diseases'	21966	2016/2/14
#1	Search stroke	243924	2016/2/14	#1	Search 'stroke'/de OR 'stroke'	362947	2016/2/14

Supplementary Table II Reasons for study exclusion.

News, letters, comments, reviews, meta-analysis, or conference abstract.

1. Zhao M, Liu W. Nut consumption decreases risk of some diseases. Am J Clin Nutr. 2014;100(3):982.
2. Addala S, Banerjee S, Ames D. Lifestyle habits and risk of stroke. Audit of medical notes may provide direction. BMJ. 2009;338:b1310
3. Cheng TO. Influence of dietary patterns on stroke risk in China. Stroke. 2005;36(2):228.
4. Ros E. Nuts and cardiovascular disease. Ann Nutr Metab. 2013;62:3.
5. Zhang Z, Xu G, Wei Y, Zhu W, Liu X. Nut consumption and risk of stroke. Eur J Epidemiol. 2015 Mar;30(3):189-96.
6. Zhou D, Yu H, He F, Reilly KH, Zhang J, Li S, Zhang T, Wang B, Ding Y, Xi B. Nut consumption in relation to cardiovascular disease risk and type 2 diabetes: a systematic review and meta-analysis of prospective studies. Am J Clin Nutr. 2014;100(1):270-7.
7. Afshin A, Micha R, Khatibzadeh S, Mozaffarian D. Consumption of nuts and beans and risk of incident coronary heart disease, stroke, and diabetes mellitus: A systematic review and meta-analysis. Circulation 2013;127.
8. Djousse L, Petrone A, Gaziano J. Nut consumption is associated with a lower risk of death among us male physicians. Circulation 2014;129: AP067.
9. Afshin A, Micha R, Khatibzadeh S, Mozaffarian D. Consumption of nuts and legumes and risk of incident ischemic heart disease, stroke, and diabetes: a systematic review and meta-analysis. Am J Clin Nutr. 2014;100(1):278-88.
10. Luo C, Zhang Y, Ding Y, Shan Z, Chen S, Yu M, Hu FB, Liu L. Nut consumption and risk of type 2 diabetes, cardiovascular disease, and all-cause mortality: a systematic review and meta-analysis. Am J Clin Nutr. 2014;100(1):256-69.
11. Grosso G, Yang J, Marventano S, Micek A, Galvano F, Kales SN. Nut consumption on all-cause, cardiovascular, and cancer mortality risk: a systematic review and meta-analysis of epidemiologic studies. Am J Clin Nutr. 2015;101(4):783-93.
12. Mayhew AJ, de Souza RJ, Meyre D, Anand SS, Mente A. A systematic review and meta-analysis of nut consumption and incident risk of CVD and all-cause mortality. Br J Nutr. 2016;115(2):212-25.
13. Nus M, Ruperto M, Sánchez-Muniz FJ. Nuts, cardio and cerebrovascular risks. A Spanish perspective. Arch Latinoam Nutr. 2004;54(2):137-48.
14. Ros E. Nuts and CVD. Br J Nutr. 2015;113 Suppl 2:S111-20.
15. Boden-Albala B, Sacco RL. Lifestyle factors and stroke risk: exercise, alcohol, diet, obesity, smoking, drug use, and stress. Curr Atheroscler Rep. 2000;2(2):160-6.
16. Boden-Albala B, Southwick L, Carman H. Dietary interventions to lower the risk of stroke. Curr Neurol Neurosci Rep. 2015;15(4):15.
17. Kivipelto, M. Lifestyle related factors in stroke and dementia. J Neurol Sci.2009;283: 242-243

18. Hankey GJ. Risk factor management to prevent stroke. Adv Neurol. 2003;92:179-85.
19. Petrović G. Risk factors for development of cerebrovascular stroke. Med Pregl. 2000;53(3-4):207-14.
20. Jia Q, Liu L, Wang Y. Risk factors and prevention of stroke in the Chinese population. J Stroke Cerebrovasc Dis. 2011;20(5):395-400.
21. Sacco RL. Risk factors, outcomes, and stroke subtypes for ischemic stroke. Neurology. 1997;49(5 Suppl 4):S39-44.
22. Srinath Reddy K, Katan MB. Diet, nutrition and the prevention of hypertension and cardiovascular diseases. Public Health Nutr. 2004;7(1A):167-86.
23. Zhang Y, Hu G. Dietary Pattern, Lifestyle Factors, and Cardiovascular Diseases. Current nutrition reports. 2012;1:64-72.
24. [No authors listed]. Lifestyle modifications help reduce stroke risk. Harv Health Lett. 2015;40(3):8.
25. [No authors listed]. Reduce your risk of silent strokes. Exercise, eat a healthy diet, and manage blood pressure and cholesterol to lower your odds. Harv Health Lett. 2015;40(5):3.
26. Bertke KA, Keyserling T. Dietary habits and foods consumed in the stroke belt. J Am Geriatr Soc. 2013;61:S226.

Reporting data about dietary patterns or no data about nut consumption

27. Kagan A, Popper JS, Rhoads GG, Yano K. Dietary and other risk factors for stroke in Hawaiian Japanese men. Stroke. 1985;16(3):390-6.
28. Bernhardt R, Feng Z, Wang Z, Deng Y, Schettler G. Risk factors for atherosclerotic vascular diseases in the People's Republic of China. Monogr Atheroscler. 1986;14:35-9.
29. Dalmeijer GW, Struijk EA, van der Schouw YT, Soedamah-Muthu SS, Verschuren WM, Boer JM, Geleijnse JM, Beulens JW. Dairy intake and coronary heart disease or stroke--a population-based cohort study. Int J Cardiol. 2013;167(3):925-9.
30. Kondo 1, Ojima T, Nakamura M, Hayasaka S, Hozawa A, Saitoh S, Ohnishi H, Akasaka H, Hayakawa T, Murakami Y, Okuda N, Miura K, Okayama A, Ueshima H; NIPPON DATA80 Research Group. Consumption of dairy products and death from cardiovascular disease in the Japanese general population: the NIPPON DATA80. J Epidemiol. 2013;23(1):47-54.
31. Kurth T, Moore SC, Gaziano JM, Kase CS, Stampfer MJ, Berger K, Buring JE. Healthy lifestyle and the risk of stroke in women. Arch Intern Med. 2006;166(13):1403-9.
32. Fung TT, Stampfer MJ, Manson JE, Rexrode KM, Willett WC, Hu FB. Prospective study of major dietary patterns and stroke risk in women. Stroke. 2004;35(9):2014-9.
33. Hlebowicz J, Persson M, Gullberg B, Sonestedt E, Wallström P, Drake I, Nilsson J, Hedblad B, Wirfält E. Food patterns, inflammation markers and incidence of cardiovascular disease: the Malmo Diet and Cancer study. J Intern Med. 2011;270(4):365-76.
34. Zhang Y, Tuomilehto J, Jousilahti P, Wang Y, Antikainen R, Hu G. Lifestyle factors and antihypertensive treatment on the risks of ischemic and hemorrhagic

stroke. Hypertension. 2012;60(4):906-12.

35. Larsson SC, Åkesson A, Wolk A. Primary prevention of stroke by a healthy lifestyle in a high-risk group. Neurology. 2015;84(22):2224-8
36. Larsson SC, Akesson A, Wolk A. Overall diet quality and risk of stroke: a prospective cohort study in women. Atherosclerosis. 2014;233(1):27-9.
37. Larsson SC, Akesson A, Wolk A. Healthy diet and lifestyle and risk of stroke in a prospective cohort of women. Neurology. 2014;83(19):1699-704.
38. Larsson SC, Virtamo J, Wolk A. Dairy consumption and risk of stroke in Swedish women and men. Stroke. 2012;43(7):1775-80.
39. Larsson SC, Virtamo J, Wolk A. Dietary protein intake and risk of stroke in women. Atherosclerosis. 2012;224(1):247-51.
40. Yamori Y, Kihara M, Fujikawa J, Soh Y, Nara Y, Ohtaka M, Horie R, Tsunematsu T, Note S, Fukase M. Dietary risk factors of stroke and hypertension in Japan -- Part 1: Methodological assessment of urinalysis for dietary salt and protein intakes. Jpn Circ J. 1982;46(9):933-8.
41. Yamori Y, Kihara M, Fujikawa J, Soh Y, Nara Y, Ohtaka M, Horie R, Tsunematsu T, Note S, Kukase M. Dietary risk factors of stroke and hypertension in Japan -- Part 2: Validity of urinalysis for dietary salt and protein intakes under a field condition. Jpn Circ J. 1982;46(9):939-43.
42. Pashiri MT, Koh WP, Pan A. Dairy intake and risk of cardiovascular mortality in Singapore Chinese adults. Circulation 2015;132: SUPPL. 3.
43. Sonestedt E, Wirfält E, Wallström P, Gullberg B, Orho-Melander M, Hedblad B. Dairy products and its association with incidence of cardiovascular disease: the Malmö diet and cancer cohort. Eur J Epidemiol. 2011;26(8):609-18.
44. Wallström P, Sonestedt E, Hlebowicz J, Ericson U, Drake I, Persson M, Gullberg B, Hedblad B, Wirfält E. Dietary fiber and saturated fat intake associations with cardiovascular disease differ by sex in the Malmö Diet and Cancer Cohort: a prospective study. PLoS One. 2012;7(2):e31637.
45. Lin PH, Yeh WT, Svetkey LP, Chuang SY, Chang YC, Wang C, Pan WH. Dietary intakes consistent with the DASH dietary pattern reduce blood pressure increase with age and risk for stroke in a Chinese population. Asia Pac J Clin Nutr. 2013;22(3):482-91.
46. Lindenstrøm E, Boysen G, Nyboe J. Lifestyle factors and risk of cerebrovascular disease in women. The Copenhagen City Heart Study. Stroke. 1993;24(10):1468-72.
47. Lindenstrøm E, Boysen G, Nyboe J. Risk factors for stroke in Copenhagen, Denmark. II. Life-style factors. Neuroepidemiology. 1993;12(1):43-50.
48. Meng L, Maskarinec G, Lee J, Kolonel LN. Lifestyle factors and chronic diseases: application of a composite risk index. Prev Med. 1999;29(4):296-304.
49. Zhang Y, Tuomilehto J, Jousilahti P, Wang Y, Antikainen R, Hu G. Lifestyle factors on the risks of ischemic and hemorrhagic stroke. Arch Intern Med. 2011 14;171(20):1811-8.
50. Zhang Y, Tuomilehto J, Jousilahti P, Wang Y, Antikainen R, Hu G. Lifestyle factors and antihypertensive treatment on the risks of ischemic and hemorrhagic stroke. Hypertension. 2012;60(4):906-12.
51. Estruch R, Ros E, Salas-Salvadó J, Covas MI, Corella D, Arós F, Gómez-Gracia E, Ruiz-Gutiérrez V, Fiol M, Lapetra J, Lamuela-Raventos RM, Serra-Majem L,

Pintó X, Basora J, Muñoz MA, Sorlí JV, Martínez JA, Martínez-González MA; PREDIMED Study Investigators. Primary prevention of cardiovascular disease with a Mediterranean diet. N Engl J Med. 2013;368(14):1279-90.

Shorter follow-up than others on the same cohort

52. He K, Merchant A, Rimm EB, Rosner BA, Stampfer MJ, Willett WC, Ascherio A. Dietary fat intake and risk of stroke in male US healthcare professionals: 14 year prospective cohort study. BMJ. 2003 Oct 4;327(7418):777-82.
53. Bernstein AM, Pan A, Rexrode KM, Stampfer M, Hu FB, Mozaffarian D, Willett WC. Dietary protein sources and the risk of stroke in men and women. Stroke. 2012 Mar;43(3):637-44.

Supplementary Table III General characteristic of included studies.

References	Country	Study name	Sex	Age at baseline	Follow-up, year	No. of cases	No. of participants	Stroke diagnosed	Exposure (Ht vs. Lt)	RR(95% CI)	Adjusted or matched variables
Yochum et al., 2000 [6]	United States	Iowa Women's Health Study	F	55-69	11	215	34,492	ICD-9 430-438	>4 times/month vs. <0	0.73(0.41-1.29)	Age, total energy intake, BMI, WHR, HBP, diabetes, alcohol intake, education, E(P)RT, marital status, smoking, physical activity, intakes of cholesterol, saturated fat, fish, vitamin C, carotenoids, dietary fiber, and whole grains
Djoussé et al., 2010 [8]	United States	The Physicians' Health Study I	M	40.7-86.7	21.1	1,424	21,078	Medical records	7 times/week vs. 0	1.07(0.79–1.46)	Age, aspirin assignment, BMI, HBP, diabetes, alcohol intake, education, smoking, physical activity, atrial fibrillation, coronary heart disease, dairy consumption, breakfast cereal, red meat, fish, fruit and vegetable intake.
Yaemsiri et al., 2012 [10]	United States	The Women's Health Initiative Observational Study	F	50-79	8	1049	87,025	Medical records	1 medium servings/d vs. 0	0.89 (0.66-1.20)	Age, race, education, family income, smoking, hormone replacement therapy use, total metabolic equivalent task hours per week, alcohol intake, history of coronary heart disease, history of atrial fibrillation, history of diabetes, aspirin use, use of antihypertensive medication, use of cholesterol-lowering medication, BMI, systolic blood pressure, total energy intake, dietary vitamin E, fruits and vegetable intake, and fiber
Bao et al., 2013 [12]	United States	The Nurses' Health Study	F	30-55	30	873	76,464	ICD- 9 430-438	≥5 servings/week vs. 0	1.05 (0.73–1.52)	Age, race, BMI, physical activity, smoking, alcohol consumption, physical examination for screening purposes, multivitamin use, current aspirin use, family history of diabetes mellitus, family history of myocardial infarction, family history of cancer, history of diabetes mellitus, history of hypertension, history of hypercholesterolemia, intake of total energy, red/processed meat, fruits, and vegetables, and menopausal status and hormone use.
Bao et al., 2013 [12]	United States	The Health Professionals Follow-Up Study	M	40-75	24	687	42,498	ICD-9 430-438	≥5 servings/week vs. 0	0.78 (0.58–1.06)	Age, race, BMI, physical activity, smoking, alcohol consumption, physical examination for screening purposes, multivitamin use, current aspirin use, family history of diabetes mellitus, family history of myocardial infarction, family history of cancer, history of diabetes mellitus, history of hypertension, history of hypercholesterolemia, intake of total energy, red/processed meat, fruits, and vegetables.
Haring et al., 2015 [13]	United States	The Atherosclerosis Risk in Communities Study	M,F	45-64	22.7	699	11601	Medical records	1 serving/d vs. 0	1.00(0.77–1.31)	Age, sex, race, study center, total energy intake, smoking, education, HBP, use of antihypertensive medication, high density lipoprotein cholesterol, total cholesterol, use of lipid lowering medication, BMI, WHR, alcohol intake, physical activity, carbohydrate intake, fiber intake, fat intake, and magnesium intake
Gopinath et al., 2015 [14]	Australia	The Blue Mountains Eye Study	M,F	>49	15	430	2893	ICD-9 and 10 Revision	Tertile 3 vs. Tertile 1	0.88(0.60-1.29)	Age, sex, qualifications, total diet score, BMI, smoking, alcohol intake, self-rated health, walking disability, HBP, diabetes, doctor-diagnosed history of cancer, angina, stroke and/or acute myocardial infarction.
Hshieh et al., 2015 [15]	United States	The Physicians' Health Study (PHS) I and II	M	66.6±9.3	9.6	14	20,742	Medical records	≥5 servings/week vs. <1 serving/month	0.64(0.32-1.30)	Age, BMI, alcohol intake, smoking, exercise, calories, saturated fat consumption, fruit/vegetable consumption, red meat consumption, prevalent diabetes, and hypertension.
Bonaccio et al., 2015 [16]	Italy	The Moli-sani study	M,F	--	4.3	19	19,386	ICD-9 430-438	Nut intake vs. no intake	1.01(0.37-2.76)	Age, sex, education, smoking, physical activity, BMI, energy intake, Mediterranean diet score without nuts, C-reactive protein platelet count, and the neutrophil to lymphocyte ratio.
Luu et al., 2015 [17]	United States	The Southern Community Cohort Study,	M,F	40-79	5.4	217	71,764	ICD-10 I61-64	Q5 vs. Q1	African-IS: 0.89 (0.45-1.74), African-HS: 1.37(0.67-2.80); European-IS: 0.47(0.12-1.76); European-HS: 0.62(0.12-3.26)	Age, sex, education, occupation, household income, marital status, smoking, alcohol intake, BMI, physical activity, vitamin supplement use, Charlson Comorbidity Index, metabolic conditions, total energy intake, red meat intake, chicken intake, seafood intake, vegetable intake, and fruit intake.
Luu et al., 2015[17]	China	The Shanghai Women's Health Study	F	40-70	12.2	706	74,741	ICD-9 430-435	Q5 vs. Q1	IS:0.72(0.51-1.03); HS:0.77 (0.55-1.07)	Age, education, occupation, household income (SMHS) or income per capita (SWHS), smoking status, alcohol intake, BMI, physical activity, regular tea consumption, Charles comorbidity index, metabolic conditions, total energy intake, red meat intakes, chicken/duck intake, seafood intake, vegetable intake, and fruit intake.
Luu et al., 2015 [17]	China	The Shanghai Men's Health Study	M	40-74	6.5	479	61,480	ICD-9 430-435	Q5 vs. Q1	IS:0.79(0.54-1.14); HS:0.80 (0.55-1.16)	Age, education, occupation, household income (SMHS) or income per capita (SWHS), smoking status, alcohol intake, BMI, physical activity, regular tea consumption, Charles comorbidity index, metabolic conditions, total energy intake, red meat intakes, chicken/duck intake, seafood intake, vegetable intake, and fruit intake.
den Brandt et al., 2015[18]	The Netherland	The Netherlands Cohort Study	M,F	55-69	10	565	120,852	ICD-9 430-438	> 10g vs. 0	0.76(0.56–1.02)	Age, sex, smoking, history of physician-diagnosed hypertension, diabetes, body height, BMI, non-occupational physical activity, education, alcohol intake, vegetables and fruit, energy, use of nutritional supplements, and postmenopausal HRT (women).
Di Giuseppe et al., 2015 [19]	German	The European Prospective Investigation into the Cancer and Nutrition Potsdam Study	M,F	F: 49.2 M: 52.5	8.3	288	26,285	ICD-10 I63, ICD-10 I60, ICD-10 I61, ICD-10 I64	> 1 portion per week vs. 1/2 portion per week	1.37(0.92–2.05)	Age, sex, BMI, waist circumference, prevalent hypertension, hyperlipidemia, diabetes, smoking, educational attainment and sport activity, alcohol intake, red meat, whole-grain breads, fruit, vegetable, fish, cakes and cookies, confectionary, fried potatoes, other beverages and total energy.

HS, hemorrhagic strokes; IS, ischemic strokes; M, male; F, female; Ht, highest; Lt, lowest; HRT, hormone replacement therapy, ERT, estrogen replacement therapy, SMHS, Shanghai Men's Health Study; SWHS, Shanghai Women's Health Study.

Supplementary Table IV Assessment of bias risk of include studies

Study	Selection				Comparability[1]	Outcome			
	Representativeness of the exposed cohort	Selection of the unexposed cohort	Ascertainment of exposure	Outcome of interest not present at start of study	Control for important factor or additional factor	Assessment of outcome	Follow-up long enough for outcomes to occur[2]	Adequacy of follow-up of cohorts[3]	Total quality scores
Yochum et al., 2000 [6]	☆	☆	☆	☆	☆	☆	☆	☆	8
Djoussé et al., 2010 [8]	-	☆	☆	☆	☆	☆	☆	☆	7
Yaemsiri et al., 2012 [10]	☆	☆	☆	☆	☆	☆	-	☆	7
Bao et al., 2013 [12] NHS	-	☆	☆	☆	☆	☆	☆	☆	7
Bao et al., 2013 [12] HPFS	-	☆	☆	☆	☆	☆	☆	☆	7
Haring et al., 2015 [13]	☆	☆	☆	☆	☆	☆	☆	☆	8
Gopinath et al., 2015[14]	☆	☆	☆	☆	☆	☆	☆	☆	8
Hshieh et al., 2015 [15]	-	☆	☆	☆	☆	☆	-	☆	6
Bonaccio et al., 2015 [16]	☆	☆	☆	☆	☆	☆	-	☆	7
Luu et al., 2015 [17] SCCH	☆	☆	☆	☆	☆	☆	-	☆	7
Luu et al., 2015[17] SWHS	☆	☆	☆	☆	☆	☆	☆	☆	8
Luu et al., 2015[17] SMHS	☆	☆	☆	☆	☆	☆	-	☆	7
den Brandt et al., 2015[18]	☆	☆	☆	☆	☆	☆	☆	☆	8
Di Giuseppe et al., 2015 [19]	☆	☆	☆	☆	☆	☆	-	☆	7

[1] According to the Newcastle-Ottawa Scale guideline, a maximum of two stars can be assigned for comparability. However, we adopted the guideline with some modification in present meta-analysis. Since confounder is the major concern in observational studies, no more than one star could be assigned. When the included studies provided risk estimates adjusted for more than ten covariates, one star could be assigned. Otherwise, no star could be assigned.

[2] One star could be assigned to a cohort study if they were followed up for ten follow-up years or more.

[3] A cohort study with a follow-up rate > 75% was given one star

Supplementary Table V Results of sensitivity analysis

Study excluded	Summary RR (95% CI)	Heterogeneity	
		P	I^2
Yochum et al., 2000 [6]	0.89(0.81-0.98)	0.486	0%
Djoussé et al., 2010 [8]	0.87(0.79-0.96)	0.581	0%
Yaemsiri et al., 2012 [10]	0.88(0.80-0.97)	0.453	0%
Bao et al., 2013 [12]	0.88(0.80-0.97)	0.453	0%
Haring et al., 2015 [13]	0.87(0.79-0.96)	0.526	0%
Gopinath et al., 2015 [14]	0.88(0.80-0.97)	0.453	0%
Hshieh et al., 2015 [15]	0.89(0.81-0.98)	0.516	0%
Bonaccio et al., 2015 [16]	0.88(0.80-0.97)	0.458	0%
Luu et al., 2015 African-IS [17]	0.88(0.80-0.97)	0.453	0%
Luu et al., 2015 African-HS [17]	0.88(0.80-0.96)	0.567	0%
Luu et al., 2015 European-IS [17]	0.89(0.81-0.97)	0.518	0%
Luu et al., 2015 European-HS [17]	0.88(0.81-0.97)	0.466	0%
Luu et al., 2015 Asia-IS [17]	0.90(0.82-1.00)	0.552	0%
Luu et al., 2015 Asia-HS [17]	0.90(0.82-1.00)	0.557	0%
Brandt et al., 2015 [18]	0.90(0.81-0.99)	0.535	0%
Di Giuseppe et al., 2015 [19]	0.86(0.78-0.95)	0.826	0%

Supplementary Table VI The quality of evidence based on GRADE system.

Exposure	Quality assessment								Quality
	No. of studies	Design	Risk of bias	Inconsistency	Indirectness	Imprecision	Publication bias	Other considerations	
Nut intake	11	Cohort study	Not serious	No serious (I^2=0%)	No serious	No serious	undetected	dose response gradient	moderate

Supplementary Table VII Comparison with Previous Meta-analyses

	Afshin et al. 2014 [2]	Shi et al. 2014	Zhou et al. 2014 [20]	Zhang et al. 2015 [21]	Mayhew et al. 2016 [3]	The present study
Number of studies (References)	4 (7-9, 12)	3 (6, 8, 9)	3 (6, 8, 9)	6 (6-10, 12)	6 (9, 10, 12, 17-19)	11 (6, 8, 10, 12-19)
Search date updated	December 25, 2013	January 31, 2014	October 10, 2013	June 2014	July 2015	February 14, 2016
Study Quality						
Main finding (H vs. L) RR with 95% CI	NR	0.90(0.81-0.99)	0.87(0.74-1.03)	Total stroke: 0.90(0.83-0.98); Stroke mortality: 0.86(0.69-1.06)	Total stroke: 1.05(0.95-1.61); Stroke mortality: 0.83(0.69-1.00)	Total stroke: 0.88(0.80-0.97); Stroke mortality: 0.81(0.72-0.91)
Dose-response analysis: linear or nonlinearity?	Linear or nonlinearity? 0.89(0.74-1.05) for per 4 weekly servings	NR	Linear association without significance; 0.90(0.71-1.14) for one serving/day	Linear association without significance; 0.94(0.82-1.08) for one serving/day	Linear or nonlinearity?; 0.85(0.55- 1.31) for per 4 weekly servings	Nonlinearity; 0.86(0.79-0.94) for 12 grams of nut per day.
Subgroup analysis	Stroke subtypes	Gender, location, time of follow-up, outcome, number of cases, and adjustments	NR	Gender, location, stroke subtypes, time of follow-up, Sample size, publication year, Quality score.	NR	gender, location, stroke subtypes, and time of follow-up
Sensitivity analyses	NR	NR	NR	Applied	NR	Applied
Publication bias	Detected	Undetected	Undetected	Undetected	NR	Undetected
Power analysis	NR	NR	NR	NR	NR	Report (86.2%)
GRADE used for evidence	NR	NR	NR	NR	Low	Moderate

NR: not report.

Shi, Z.Q. et al. Consumption of nuts and legumes and risk of stroke: a meta-analysis of prospective cohort studies. *Nutr Metab Cardiovasc* Dis **24**, 1262-71 (2014).

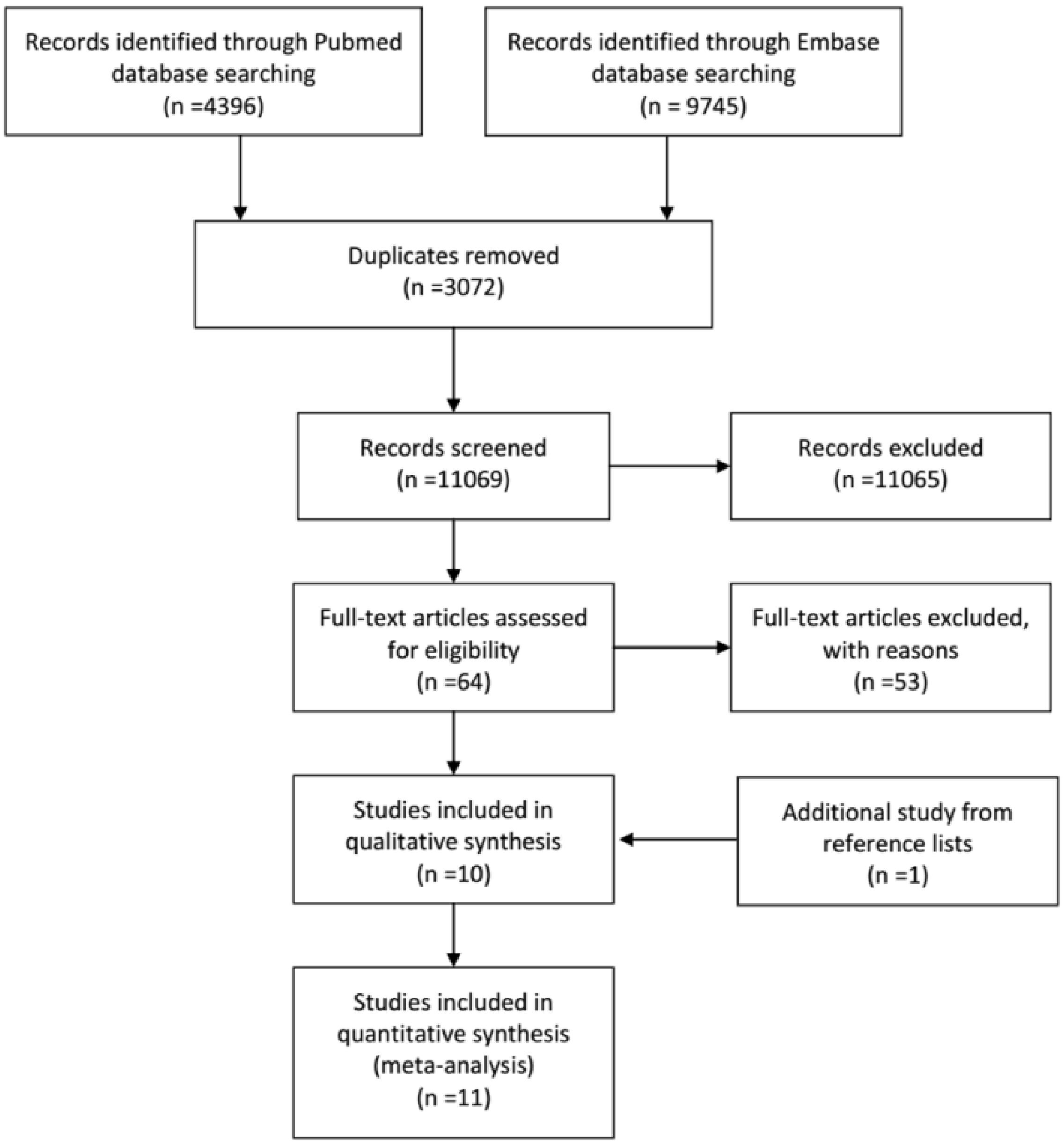

Supplementary Figure I Flow Diagram

Supplementary Appendix 1 SAS code for Power Calculations

Power calculations: The methodology used is described by Cafri 2009[341] and corresponding macro was obtained from the Supplementary Material in Cafri 2009[34]. The macro used and results are below:

```
✧✧ Power calculation for meta-analysis of nut ✧✧
data nut;
input es v;
cards;
-0.3147 0.0504
0.0677 0.0292
-0.1165 0.0190
-0.1165 0.0176
0 0.0190
-0.1278 0.0310
-0.4463 0.0625
0.0100 0.3717
-0.1165 0.1083
0.3148 0.2952
-0.7550 0.1750
-0.4780 0.6416
-0.2614 0.0104
-0.2614 0.0099
-0.2744 0.0138
0.3148 0.0831
;
run;
%metapower(test='M', model='random', raw_data='yes', alpha=.05, tau2=99, heterogeneity=99, n1=99, n2=99, k=99, eff_type='rr', T=-0.127833372, Dataset= nut, B=NA,v=v,
x=NA, es=es, p=NA, weight=NA);

run;
```

```
---------------------Meta-Analysis Power Macro---------------------
Test of Mean Effect Size

Model = random
Effect Size Metric = rr
Raw data provided= Yes
Mean Effect Size = -0.127833
Number of Studies = 16
Random Effects Variance= 0
Sampling Variance = 0.0017585
Alpha = 0.05
Estimated Power of Test (One-Tailed) = 1.3443E-6
Estimated Power of Test (Two-Tailed) = 0.8618045
-------------------------------------------------------------------
```
